# A Thrombotic Thrombocytopenic Purpura-Like Syndrome in HIV and Streptococcus pneumoniae Meningitis

**DOI:** 10.7759/cureus.30966

**Published:** 2022-11-01

**Authors:** Julia L Boland, Claire Valburg, Hayley Rogers, Adrienne N Poon

**Affiliations:** 1 Internal Medicine, George Washington University School of Medicine and Health Sciences, Washington, DC, USA

**Keywords:** human immunodeficiency virus infection, therapeutic plasmapheresis, adamts13 activity, thrombotic microangiopathy, streptococcus pneumoniae meningitis, thrombotic thrombocytopenic purpura (ttp)-like syndrome

## Abstract

Thrombotic thrombocytopenic purpura (TTP) is a disorder characterized by the formation of diffuse thromboses in small blood vessels, which can result in neurological and renal impairment, fever, and purpura, among additional sequelae. TTP-like syndromes are disease processes that have similar signs and symptoms as TTP but without a severe deficiency in ADAMTS13 levels. We present a case of a young male with advanced human immunodeficiency virus (HIV) and *Streptococcus pneumoniae *meningitis presenting with a thrombotic microangiopathy (TMA). Although his ADAMTS13 level was not suggestive of TTP, at 54.4% (normal low ADAMTS13: >66.8% activity; severe ADAMTS13 deficiency: *≤*10% activity), he improved only after plasmapheresis was initiated, supporting a diagnosis of a TTP-like syndrome likely due to his streptococcal meningitis. We discuss the importance of treating patients with TTP-like syndromes and advanced HIV with highly active antiretroviral therapy (HAART). We also highlight the increased prevalence of TMA and TTP among HIV patients and that many of these patients do not have a severe deficiency in levels of serum ADAMTS13.

## Introduction

Idiopathic thrombotic thrombocytopenic purpura (TTP) is characterized by thrombocytopenia, microangiopathic hemolytic anemia, organ ischemia, and a reduction in ADAMTS13 [1]. Thrombotic microangiopathy (TMA), on the other hand, describes the clinical features of TTP without a decrease in ADAMTS13 level or ADAMTS13 autoantibodies [1]. TTP is relatively rare in the general population; however, it has been shown that the rates of TTP-like syndromes in human immunodeficiency virus (HIV) patients have been estimated to be up to 15-40 times higher than in HIV-negative patients [2]. Some case reports and meta-analyses have found that many HIV-positive TTP patients have normal levels of ADAMTS13 and no autoantibodies to ADAMTS13 [3,4]. An analysis of 20 HIV-affected patients with TTP-like syndrome showed that all four of the patients with cluster of differentiation 4 (CD4) counts less than 100 cells/IL had normal ADAMTS13 levels [4]. HIV/AIDS patients may present with a TTP-like syndrome despite normal serum ADAMTS13 levels and no ADAMTS13 autoantibodies. Our case describes a patient with bacterial meningitis triggering TMA, a TTP-like syndrome with normal ADAMTS13 levels.

## Case presentation

A male in his 40s with a past medical history of poorly controlled HIV was brought into the emergency department (ED) from a homeless shelter for altered mental status and unresponsiveness. He was noncompliant with his antiretroviral medications but otherwise had no other past medical history. His family history was noncontributory. His vital signs on admission included a fever of 101.8°F, blood pressure of 132/96 mmHg, tachycardia of 145 beats per minute (bpm), tachypnea of 25 respirations per minute, and an oxygen saturation of 96% on room air. On physical examination, he was cachectic and obtunded. He had no rashes, lesions, or petechiae. His cardiac examination showed tachycardia without murmurs.

His initial laboratory tests revealed a hemoglobin of 12.6 g/dL, platelet count of 99,000/mcL, international normalized ratio (INR) of 2.02, reticulocyte count of 0.4%, haptoglobin of <20 mg/dL, lactate dehydrogenase (LDH) of 794 U/L, lactate of 4.2 mg/dL, and creatinine of 1.2 mg/dL, as shown in Table 1. His HIV laboratory assessment was notable for a CD4 count of 3 cells/m^3^ and an HIV viral load of 965,000 copies/mL. A lumbar puncture was performed in the emergency room, which demonstrated an opening pressure of 53 cm H_2_O and a white blood cell count of 19,000/µL (99% polymorphonuclear leukocytes). A cerebrospinal fluid (CSF) culture showed gram-positive cocci, which was found to be *Streptococcus pneumoniae*. His CSF cryptococcus results were negative. Serum toxoplasmosis and hepatitis antibodies were negative. He was admitted to the intensive care unit for *Streptococcus pneumoniae* meningitis and bacteremia.

**Figure 1:**
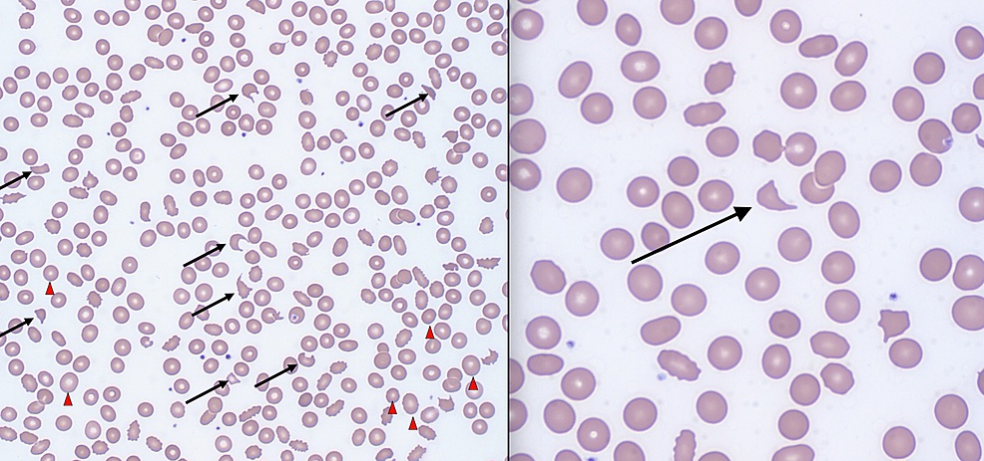
Peripheral Blood Smear Peripheral blood smear with schistocytes (arrows) and anisocytosis (arrowheads) (left: ×100; right: ×50)

During his hospitalization, he was noted to have a downtrending platelet count of less than 20,000/mcL and an associated hemoglobin drop of 5.4 g/dL. Laboratory tests for hemolysis were notable for a haptoglobin of <20 mg/dL, an LDH of 2,142 U/L, and multiple schistocytes on a peripheral smear (Figure 1, arrow). His INR was 1.05, and his ADAMTS13 level was 54.4% (normal low: >66.8%). His direct antiglobulin test and serotonin release assay were both negative.

**Figure 2:**
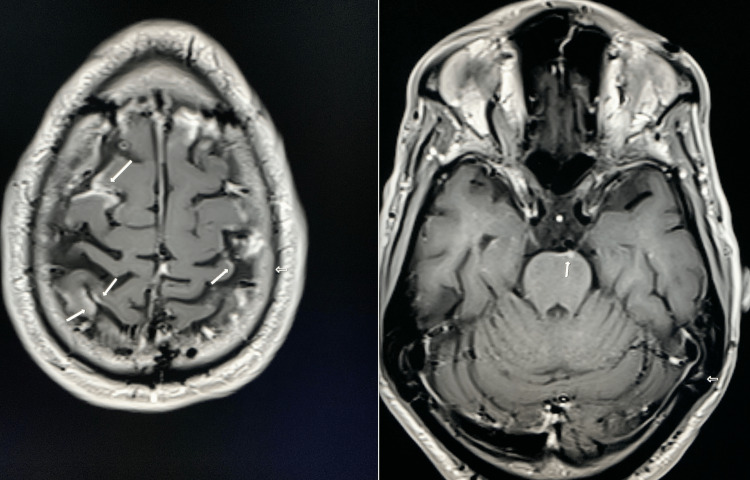
MRI Brain MRI brain demonstrating ependymal enhancement involving bilateral occipital horns (left) and leptomeningeal enhancement involving bilateral frontoparietal sulci at the vertex and ventral surface of the pons (right), representing meningitis with ventriculitis

A transthoracic echocardiogram was normal without vegetations. He had an MRI brain, which showed abnormal leptomeningeal enhancement involving bilateral frontoparietal sulci at the vertex and ventral surface of the pons and ependymal enhancement involving bilateral occipital horns, representing meningitis with ventriculitis (Figure 2).

No bleeding or thrombosis was initially noted on imaging studies. However, later imaging revealed a provoked nonocclusive thrombus in his right internal jugular vein in the location of a central line. An ultrasound of his spleen was unremarkable. Because of the high likelihood of TTP, urgent initiation of plasmapheresis was performed. He received four rounds of plasmapheresis in the intensive care unit. For his bacterial meningitis, he was initially treated with ceftriaxone, vancomycin, and dexamethasone. This regimen was narrowed to ceftriaxone 2 g daily. Because of his prolonged hospital course and disposition to go to rehab, the decision was made to start antiretrovirals with bictegravir 50 mg, emtricitabine 200 mg, and tenofovir alafenamide 25 mg daily, along with darunavir 800 mg and cobicistat 150 mg daily. He was started on antiretrovirals three weeks after presenting to the ED, with a plan to continue on discharge. He was also started on trimethoprim 80 mg and sulfamethoxazole 400 mg daily for *Pneumocystis jiroveci* pneumonia (PJP) prophylaxis.

After three rounds of plasmapheresis, the patient’s laboratory testing demonstrated a platelet count of 117,000/mcL, hemoglobin of 11.0 g/dL, lactate dehydrogenase (LDH) of 1,303 U/L, and haptoglobin of 105 mg/dL. After a fourth round of plasmapheresis, he showed sustained clinical response, with platelet counts remaining stable within normal limits, as shown in Table 1.

**Table 1:** Laboratory Results The patient’s laboratory test results initially, one week after he presented to the emergency department, and after plasmapheresis MCV: mean corpuscular volume; INR: international normalized ratio; LDH: lactate dehydrogenase

	Initial Laboratories	One Week Post Presentation	Post-plasmapheresis
Hemoglobin (g/dL)	12.6	5.1	9.5
MCV (fL)	88.9	91.7	28.9
Platelets (100,000/mcL)	99	<20	117
INR	2.02	1.18	1.06
Reticulocyte count (%)	0.4 (L)	0.8	1.2
Haptoglobin (mg/dL)	<20	51	105
LDH (U/L)	794	887	1,303
Lactate (mg/dL)	4.2->1.0	Not done	Not done
Creatinine (mg/dL)	1.2	1	0.9

During his hospitalization, his HIV responded to antiretroviral treatment, with a repeat serum HIV-1 RNA by polymerase chain reaction (PCR) of 320 copy/mL (from 965,000 copy/mL on admission) and a CD4 count of 63 cells/mcL (from 3 cells/mcL on admission) over the course of one month. His mental status gradually returned to his baseline over his hospitalization. However, he suffered recurrent persistent headaches, attributed to the sequela of meningitis. His headaches responded with naproxen as needed. A follow-up appointment was made for the patient with the infectious disease department; however, the patient was lost to follow-up. As of one year after discharge from the hospital, the patient has been lost to follow-up.

## Discussion

TTP-like syndromes have been associated with different presenting factors than classical TTP, such as the involvement of the lungs, liver, and pancreas. Additionally, patients with TTP-like syndromes may have less schistocytes on peripheral blood smear [5]. However, despite these differences in presentation, patients still respond well to plasma exchange therapy, and recognizing TTP-like syndromes is critical for timely and potentially lifesaving treatment [6].

HIV has been associated with TTP-like syndromes and is more common in patients with advanced disease and low CD4 lymphocyte counts [3]. One hypothesis of the pathophysiology of TTP-like syndromes in HIV is that the elevated inflammatory markers increase the endothelial release of von Willebrand factor and inhibit the synthesis of ADAMTS13, resulting in the buildup of uncleaved von Willebrand factor [7]. Another theory is that endothelial damage by HIV/AIDS causes a buildup of thrombin and the consumption of ADAMTS13, which leads to thrombus formation and red cell fragmentation [3]. Studies have shown that discontinuing highly active antiretroviral therapy (HAART) and hence resultant uncontrolled HIV can lead to a relapse in TTP [8]. Therefore, it is critical to treat patients with TTP-like syndromes and advanced HIV with HAART.

Our patient presented with thrombocytopenia and anemia, along with rising LDH and low haptoglobin, indicating hemolysis. His negative direct Coombs antiglobulin test ruled out autoimmune hemolytic anemia, and he had a negative serotonin release assay, which decreases the likelihood of heparin-induced thrombocytopenia. He was on subcutaneous heparin for deep vein thrombosis prophylaxis during admission but did not have any history of using intravenous drugs or heparin before his hospitalization. Moreover, his peripheral blood smear revealed a large number of schistocytes, supporting the diagnosis of microangiopathic hemolytic anemia [9]. Disseminated intravascular coagulation was deemed unlikely given his normal coagulation, fibrinogen level, and lack of bleeding or thrombosis. Hemolytic uremic syndrome was also deemed unlikely due to his stable creatinine and lack of renal impairment [10].

While other classic characteristics of TTP, including fever and altered mental status, could be explained by his meningitis alone, his degree of anemia and thrombocytopenia continued to worsen despite antibiotic therapy. Although he had a platelet count, combined hemolysis variable, absence of active cancer, absence of stem-cell or solid-organ transplant, mean corpuscular volume, INR, creatinine (PLASMIC) score of 4 and his ADAMTS13 levels were normal, his sustained improvement following plasmapheresis supports the diagnosis of a TTP-like syndrome [11]. Cases of *Streptococcus pneumoniae*-associated TTP have been previously described and are likely the precipitating factor in our patient’s case [12,13]. Our patient’s clinical course suggests that providers should consider plasmapheresis as a treatment option for such individuals.

## Conclusions

Patients with HIV and AIDS are at risk of TMA and may present atypically with a TTP-like syndrome despite a lack of severe deficiency in serum ADAMTS13 level. Our case highlights that patients with HIV and TTP-like syndromes can be responsive to plasmapheresis. Therefore, it is critical to not withhold plasmapheresis in patients with concern for TTP but otherwise normal ADAMTS13 levels. Our case highlights the limitation of serum ADAMTS13 testing in advanced HIV patients with TMA and TTP-like syndromes. Moreover, we emphasize the importance of initiating HAART for advanced HIV/AIDS patients admitted to the hospital for TTP-like syndromes.

## References

[1] Joly BS, Coppo P, Veyradier A (2017). Thrombotic thrombocytopenic purpura. Blood.

[2] Louw S, Gounden R, Mayne ES (2018). Thrombotic thrombocytopenic purpura (TTP)-like syndrome in the HIV era. Thromb J.

[3] Brecher ME, Hay SN, Park YA (2008). Is it HIV TTP or HIV-associated thrombotic microangiopathy?. J Clin Apher.

[4] Gunther K, Garizio D, Nesara P (2007). ADAMTS13 activity and the presence of acquired inhibitors in human immunodeficiency virus-related thrombotic thrombocytopenic purpura. Transfusion.

[5] Chang JC (2018). TTP-like syndrome: novel concept and molecular pathogenesis of endotheliopathy-associated vascular microthrombotic disease. Thromb J.

[6] Chang JC, Newman RS (2004). Redefining the syndromes of thrombotic microangiopathy. Ther Apher Dial.

[7] Chen J, Chung DW (2018). Inflammation, von Willebrand factor, and ADAMTS13. Blood.

[8] Miller RF, Scully M, Cohen H, Roedling S, Starke R, Edwards SG, Machin SJ (2005). Thrombotic thrombocytopaenic purpura in HIV-infected patients. Int J STD AIDS.

[9] Kappler S, Ronan-Bentle S, Graham A (2017). Thrombotic microangiopathies (TTP, HUS, HELLP). Hematol Oncol Clin North Am.

[10] Villa F (2014). Syndromes of thrombotic microangiopathy. N Engl J Med.

[11] Bendapudi PK, Hurwitz S, Fry A (2017). Derivation and external validation of the PLASMIC score for rapid assessment of adults with thrombotic microangiopathies: a cohort study. Lancet Haematol.

[12] Ichikawa Y, Murata M, Aoki M (2020). Streptococcus pneumoniae-associated thrombotic microangiopathy in an immunosuppressed adult. Open Med (Wars).

[13] Walsh LF, Sherbuk JE, Wispelwey B (2021). Pneumococcal induced thrombotic thrombocytopenic purpura with features of purpura fulminans. BMJ Case Rep.

